# Immune checkpoint inhibitors: immune-related adverse events, healthcare utilization, and costs among commercial and Medicare Advantage patients

**DOI:** 10.1007/s00520-022-06826-9

**Published:** 2022-01-21

**Authors:** Krishna S. Gunturu, Timothy T. Pham, Sonali Shambhu, Michael J. Fisch, John J. Barron, David Debono

**Affiliations:** 1 Lahey Hospital and Medical Center and Lahey Health Cancer Institute, Beth Israel Lahey Health, 41 Mall Road, Burlington, MA 01805 USA; 2 HealthCore, 123 Justison St, Suite 200, Wilmington, DE 19801 USA; 3 AIM Specialty Health, Chicago, IL USA; 4 Anthem Inc, 220 Virginia Ave, Indianapolis, IN 46204 USA

**Keywords:** Immune checkpoint inhibitors, Immunotherapy, Neoplasms, Hospitalization, Adverse drug event, USA

## Abstract

**Background:**

Immune checkpoint inhibitors (ICI) are increasingly used across multiple cancer types and stages and little is known about real-world outcomes. This study sought to determine healthcare utilization, costs, immune-related adverse events (irAEs), and all-cause mortality of single-agent versus combination ICI in the USA.

**Materials and methods:**

This is a retrospective study conducted with 2016–2018 data from the HealthCore Integrated Research Database, consisting of commercial and Medicare-insured adult patients with a cancer diagnosis using ICI in the USA. Outcomes were healthcare utilization, costs, and irAEs (FDA-recognized and others) up to 1-year post-index between patients using ICI monotherapy (mono, PD-1/PD-L1 inhibitor) and combination therapy (combo, PD-1/PD-L1 with CTLA-4 inhibitors).

**Results:**

In total, 9084 patients received monotherapy and 904 patients received combo therapy. Mean age 65 years for mono and 58 years for combo. Overall, the combo arm had higher rates of FDA-recognized irAEs (67.4% vs. 45.9%), especially endocrinopathies (27.7% vs 14.7%) and dermatitis (25.9% vs. 12.4%). All-cause mortality over 1-year follow-up was similar, 30.7% in mono vs 30.8% in combo arms. The combo group had higher rates of all-cause inpatient hospitalizations (55.4% mono vs 65.6% combo) and emergency department (ED) visits (33.7% mono vs 41.4% combo). IrAE-related hospitalizations were higher in combo (55.2% vs 42.1%). IrAE-related ED visits were 15.7% mono vs 22.7% combo. This increased toxicity and health care utilization was reflected in significant differences in healthcare costs. Stark differences were seen in all-cause medical costs as well as costs related to inpatient and ED utilization and costs attributed to irAEs.

**Conclusions:**

Higher rates of irAEs, healthcare utilization, and costs occur with combination immunotherapy. As further indications are approved for combination ICI, our study highlights the real-world tradeoffs involved with combination therapy regarding burdens of toxicity and increased healthcare utilization.

**Supplementary Information:**

The online version contains supplementary material available at 10.1007/s00520-022-06826-9.

## Introduction

ICI therapy was initially approved in 2011 for melanoma. Since then, utilization of ICI has amplified across different tumor types and stages. ICI are also being approved in combination with chemotherapy, biotherapy, and targeted therapy in the treatment of various malignancies. In 2011, < 2% of cancer patients were eligible for ICI and in 2019, this number had increased to 40% [1]. Despite the promise of ICI in controlling disease and sometimes extending survival, their use is limited by immune-related adverse events (irAEs). The cost effectiveness of ICI is not fully understood and little is known about factors associated with irAEs and mortality from these agents.

To evaluate the healthcare utilization and cost in clinical practice in the USA, we retrospectively reviewed the data from HealthCore Integrated Research Database (HIRD), a large database of clinical data from a large payer program [2]. We reviewed healthcare utilization, cost, all-cause mortality, and irAEs in patients utilizing ICI as monotherapy or in combination with another ICI up to 1-year post-index. Study subjects had commercial or Medicare Advantage insurances. We hypothesize that study findings will help assess the impact of the emerging trend of combination therapy (addition of CTLA-4 inhibitors to PD-1 or PD-L1 inhibitors) regarding the adverse event risk and associated costs. These data may inform clinical guideline development and choices by patients and physicians in the context of shared decision-making.

The primary objective of this retrospective study was to evaluate healthcare utilization and cost up to 1-year post-index between patients using ICI monotherapy (PD-1/PD-L1 inhibitor) and combination therapy (PD-1/PD-L1 with CTLA-4 inhibitors) among the commercial and Medicare insured population. Secondary objectives include comparing the rates of all-cause mortality and irAEs in these same cohorts.

## Materials and methods

In this retrospective cohort analysis, we used claims from April 2016 to December 2018 to identify members with ≥ 1 claim for any ICI. The index date was assigned as first claim for the ICI. The baseline period was calculated 6 months prior to index date and the follow-up period was assigned from index date until the earliest of the end of eligibility (due to disenrollment or death), or the end of index date plus 1 year. The study cohort consisted of patients aged 18 or greater using ICI in the time period from April 2016 to December 2018 with ≥ 1 inpatient/ER or ≥ 2 outpatient claims with a cancer diagnosis within the preceding 6 months. Continuous medical and pharmacy eligibility in the 6 months prior to index date was required. No eligibility was required in the follow-up period. The eFigure in the Supplement illustrates our cohort definition procedure, including inclusion and exclusion criteria. After applying inclusion/exclusion criteria, the following two groups were created:

- *PD-1/PD-L1 inhibitor monotherapy* — members who received a PD-1 or PD-L1 inhibitor agent without a concurrent CTLA-4 inhibitor within 30 days of index PD-1/PD-L1 claim. Members were excluded if they had any of the study immunotherapy agents in the 6-month baseline period.
- *PD-1/PD-L1 inhibitor combination therapy with CTLA-4 inhibitor* — members who have used a PD-1 or PD-L1 inhibitor agent with a concurrent CTLA-4 inhibitor within 30 days of index PD-1/PD-L1 claim. Members were excluded if they had any of the study immunotherapy agents in the 6-month baseline period.

### Mortality and irAEs

Mortality at 1 year was determined from the Social Security Death Index [3], hospital claim discharge records, or health plan disenrollment records.

We used irAEs from FDA Label information [4] and other exploratory irAEs; the full list of individual irAEs and their respective ICD10 diagnosis codes are listed in eTables 1 and 2. We defined new incidence of irAEs in the post-index period as those where the irAEs did not exist in the baseline period.

### Post-index all-cause and irAE-related utilization and cost

Cost and utilization outcomes consisted of measures that included all-cause and irAE-related hospitalizations, emergency department (ED) visits, physician office visits, and physician office visits related to Hematology-Oncology providers. All-cause and irAE-related total, health plan paid, and patient paid costs for healthcare utilization were also assessed. Claims with any irAE condition in any of the diagnosis fields were considered for irAE-related utilization and cost. Costs are presented per-patient-per-month (PPPM).

### Covariates

Baseline demographic characteristics included age, sex, region, health plan type, index year, and other additional information obtained at 9-digit zip code level (e.g., race/ethnicity, median household income, Socio-economic category, and level of education). Baseline 6-month clinical characteristics consisted of the Deyo-Charlson Comorbidity Index (DCI) score excluding malignancy, weight, and claims-based ECOG score categories [5]. Important comorbidities like autoimmune conditions (rheumatoid arthritis, psoriasis/psoriatic arthritis, ulcerative colitis, Crohn’s disease, ankylosing spondylitis, juvenile chronic polyarthritis, hypo/hyper thyroid [including thyroid replacement treatment]), immune-compromised conditions (HIV, stem cell transplants), type of cancer (breast, lung, colorectal, melanoma, renal cell, bladder, head neck, gastric, esophageal, hepatocellular, lymphoma, squamous cell carcinoma, merkel cell carcinoma), and baseline irAEs were collected. Baseline medication captured included prior chemotherapy use, other immunosuppressive therapy (infliximab, vedolizumab, mycophenolate mofetil, intravenous immunoglobulin, plasmapheresis, rituximab, tacrolimus, tocilizumab, cyclosporine, cyclophosphamide, methotrexate, sulfasalazine, leflunomide), and antibiotic use (oral and IV). Other baseline measures included BMI ≥ 30, utilization, and cost.

### Statistical analysis

Patient demographic baseline and post-index unadjusted characteristics were summarized as means with standard deviations and medians with interquartile range for continuous variables and number and proportions for categorical variables. Differences in baseline characteristics were compared between monotherapy and combination therapy, using *T*-test (or Wilcoxon rank-sum test for non-normal distributions) for continuous variables and chi-square or Fisher’s exact test for categorical variables.

For adjusted analysis, we used multivariable Cox proportional hazards regression model to report the hazard ratio (HR) and 95% confidence intervals (CI) for irAE incident outcome measures. Cost and utilization outcomes were analyzed using multivariable logistic regression for binary variables, Poisson regression for count variables, and model with gamma distribution for cost variables. Regression analyses adjusted for age, sex, region, insurance plan type, index year, Deyo-Charlson Comorbidity Index score, selected cancer type (lung, melanoma, renal cell, and head/neck), prior chemotherapy, high-dose corticosteroids, and antibiotics use. All analyses were conducted using SAS Enterprise Guide, version 7.15 (SAS Institute Inc.). Statistical significance was set at a 2-sided *p* = 0.05.

## Results

In this analysis, 9084 patients received PD-1/PD-L1 monotherapy (mono) and 904 patients received combination (combo) of CTLA-4 with PD-1/PD-L1 therapy. The patient characteristics and tumor types are presented in Tables 1 and 2. In the 1-year post-index period of this study, the average follow-up time was similar between two groups (7.6 [4.4] months in mono vs. 7.8 [4.5] months in combo therapy, *p* = 0.379). We found that the majority of patients received monotherapy in comparison to combination therapy. Lung and melanoma were the leading cancer types treated in the mono arm, melanoma, and lung in combo arm. Patients treated in the combo arm were younger and male predominant compared to mono arm. The ECOG performance status was similar in both arms. The mono group had a higher rate of prior chemotherapy utilization.

**Table 1:** Demographic, clinical, and treatment characteristics in baseline

	Monotherapy (PD-1/PD-L1 inhibitor, *N* = 9084)	Combination therapy (PD-1/PD-L1 with CTLA-4 inhibitors, *N* = 904)	*p*-value
Demographic characteristics			
Age, years, mean (SD)	64.6 (12.2)	58 (11.2)	< 0.001
Female, *n* (%)	3796 (41.8)	326 (36.1)	0.001
Geographic region, *n* (%)			
Northeast	1284 (14.8)	140 (16.1)	0.013
West	2315 (26.3)	193 (22.2)	
Midwest	2441 (27.7)	229 (26.3)	
South	2770 (31.4)	308 (35.4)	
Insurance plan type, *n* (%)			
CDHP	1098 (12.1)	140 (15.5)	0.021
HMO	1962 (21.6)	201 (22.2)	
PPO	6022 (66.3)	563 (62.3)	
Other	< 10	< 10	
Medicare Advantage, *n* (%)	1355 (14.9%)	74 (8.2%)	< 0.001
Year of index, *n* (%)			
2016	1959 (21.6)	150 (16.6)	< 0.001
2017	3132 (34.5)	254 (28.1)	
2018	3993 (44.0)	500 (55.3)	
Clinical characteristics			
Deyo-Charlson Comorbidity Index Score excluding malignancy weight, mean (SD)	1.8 (1.7)	1.4 (1.6)	< 0.001
ECOG score categories (claims-based)^a^			
ECOG cat (0–1)	7825 (86.1)	785 (86.8)	0.210
ECOG cat (≥ 2)	985 (10.8)	85 (9.4)	
Missing	274 (3.0)	34 (3.8)	
Type of cancer at baseline, *n* (%)			
Breast	338 (3.7)	31 (3.4)	0.726
Lung	4695 (51.7)	205 (22.7)	< 0.001
Colorectal	344 (3.8)	21 (2.3)	0.032
Melanoma	1156 (12.7)	459 (50.8)	< 0.001
Renal cell	803 (8.8)	186 (20.6)	< 0.001
Bladder	652 (7.2)	< 10	< 0.001
Head neck	719 (8.0)	25 (2.8)	< 0.001
Gastric	160 (1.8)	7 (0.8)	0.038
Esophageal	187 (2.1)	4 (0.4)	0.001
Hepatocellular	202 (2.2)	2 (0.2)	< 0.001
Lymphoma	240 (2.6)	16 (1.8)	0.141
Squamous cell carcinoma	289 (3.2)	8 (0.9)	< 0.001
Merkel cell carcinoma	163 (1.8)	14 (1.5)	0.688
Treatment characteristics			
Prior chemotherapy, *n* (%)	3039 (33.5)	220 (24.4)	< 0.001
High-dose corticosteroids, *n* (%)	5086 (55.9)	375 (41.5)	< 0.001
Antibiotic use			
Oral use, (*n*, %)	6373 (70.2)	571 (63.2)	< 0.001
IV use, (*n*, %)	2378 (26.2)	210 (23.2)	0.059

*CDHP*, consumer-driven health plan; *CTLA-4*, cytotoxic T-lymphocyte-associated protein 4; *ECOG*, Eastern Cooperative Oncology Group; *HMO*, health maintenance organization; *PD-1*, programmed death-1; *PD-L1*, programmed death ligand-1; *PPO*, preferred provider organization.

^a^Dichotomized ECOG score produced from a model using demographic variables, healthcare service indicators, and diagnoses from administrative claims data.

**Table 2:** Incidence and risk of immune-related adverse events (irAEs) in 1-year post-index period

	Monotherapy (PD-1/PD-L1 inhibitor, *N* = 9084)	Combination therapy (PD-1/PD-L1 with CTLA-4 inhibitors, *N* = 904)	Adjusted HR (95% CI)
All-cause mortality in 1-year post-index period, *n* (%)	2786 (30.7)	278 (30.8)	1.56 (1.37–1.79)
irAEs from FDA label information, *n* (%)	4167 (45.9)	609 (67.4)	2.07 (1.88–2.28)
Pneumonitis	200 (2.2)	23 (2.5)	1.84 (1.14–2.99)
Endocrinopathies	1,336 (14.7)	250 (27.7)	2.02 (1.73–2.35)
Myocarditis	11 (0.1)	10 (1.1)	-^a^
Hepatitis^b^	700 (7.7)	155 (17.1)	2.17 (1.78–2.64)
Colitis	532 (5.9)	143 (15.8)	2.70 (2.18–3.34)
Nephritis	1304 (14)	190 (21)	1.79 (1.51–2.13)
Dermatitis	1123 (12.4)	234 (25.9)	2.13 (1.81–2.50)
Neuropathy	635 (7.0)	60 (6.6)	1.13 (0.84–1.52)
Encephalitis	31 (0.3)	< 10	3.08 (1.26–7.53)
Exploratory irAEs, *n* (%)	4690 (51.6)	567 (62.7)	1.55 (1.41–1.71)
Abdominal pain	1545 (17.0)	217 (24.0)	1.74 (1.48–2.04)
Diarrhea	1019 (11.2)	225 (24.9)	3.16 (2.51–3.99)
Infusion-related adverse events	143 (1.6)	< 10	0.70 (0.33–1.49)
Malaise and fatigue	2666 (29.3)	322 (35.6)	1.38 (1.22–1.57)
Myositis	1020 (11.2)	121 (13.4)	1.51 (1.23–1.87)
Renal failure	658 (7.2)	75 (8.3)	1.27 (0.97–1.66)

*irAE*, immune-related adverse event; *CTLA-4*, cytotoxic T-lymphocyte-associated protein 4; *HR*, hazard ratio; *PD-1*, programmed death-1; *PD-L1*, programmed death ligand-1.

^a^Not enough observations to calculate hazard ratio.

^b^Includes viral hepatitis B and hepatitis C.

Overall, the combo arm had higher rates of irAEs. The combo group also experienced higher rates of other types of incident irAEs. All-cause mortality over 1-year follow-up was similar in both arms, 30.7% in mono vs 30.8 in combo arms with HR 1.56 (1.37–1.79). The combo group had higher rates of all cause inpatient hospitalizations (55.4% in mono vs 65.6% in combo) and emergency department (ED) visits (33.7% in mono vs 41.4% in combo). IrAE-related hospitalizations in the mono arm were 42.1% vs 55.2% in combo with OR 2.17 (CI 1.87–2.53). IrAE-related ED visits were 15.7% in mono vs 22.7% in combo. Table 3 outlines significant differences in medical costs between mono and combo ICI therapy and Table 4 outlines unadjusted and adjusted means for total medical costs. Differences are noted in total medical costs, inpatient utilization costs, and ED and outpatient costs. These differences are seen in health-plan costs, but, importantly, are also seen in patient costs. The adjusted means can be quite different, leading to the results seen in Tables 3 and 4. The purpose of showing the adjusted differences was to show that the differences between combination and monotherapy were statistically significant (confidence interval does not contain 0).

**Table 3:** Utilization and cost differences between monotherapy and combination therapy

	Monotherapy (PD-1/PD-L1 inhibitor, *N* = 9084)	Combination therapy (PD-1/PD-L1 with CTLA-4 inhibitors, *N* = 904)	Adjusted OR (95% CI)
Utilization, *n* (%)			
All-cause, *n* (%)			
Inpatient hospitalization	5028 (55.4)	593 (65.6)	2.27 (1.93–2.66)
ED visit	3059 (33.7)	374 (41.4)	1.55 (1.33–1.81)
Outpatient visit	9076 (99.9)	902 (99.8)	**-**^**a**^
irAE-related, *n* (%)			
Inpatient hospitalization	3826 (42.1)	499 (55.2)	2.17 (1.87–2.53)
ED visit	1430 (15.7)	205 (22.7)	1.64 (1.36–1.97)
Outpatient visit	6653 (73.2)	729 (80.6)	1.31 (1.09–1.58)
Costs, mean $ PPPM (SD)			Adjusted difference (95% CI)
All-cause			
Total medical cost	$26,741 ($55,623)	$67,877 ($237,854)	$43,747 ($38,440–$49,427)
Plan paid	$26,354 ($55,497)	$67,309 ($236,030)	$48,443 ($42,336–$55,031)
Patient paid	$387 ($952)	$569 ($3653)	$151 ($81–$230)
Inpatient hospitalization	$8316 ($40,186)	$20,672 ($165,490)	$20,207 ($14,949–$26,527)
Plan paid	$8243 ($40,101)	$20,450 ($162,851)	$23,459 ($17,691–$30,394)
Patient paid	$73 (487)	$222 ($3553)	$119 ($90–$152)
ED visit	$286 ($1456)	$548 ($2428)	$428 ($312–$564)
Plan paid	$278 ($1439)	$537 ($2408)	$424 ($312–$556)
Patient paid	$8 ($87)	$12 ($90)	$7 ($5–$9)
Outpatient visit	$18,138 ($32,906)	$46,656 ($120,860)	$24,149 ($21,135–$27,362)
Plan paid	$17,833 ($32,862)	$46,321 ($120,850)	$24,761 ($21,494–$28,268)
Patient paid	$306 ($665)	$335 ($825)	$26 (− $21–$80)
irAE-related			
Total medical cost	$7837 ($30,841)	$22,626 ($165,487)	$19,224 ($14,911–$24,246)
Plan paid	$7748 ($30,733)	$22,376 ($162,848)	$22,964 ($18,103–$28,636)
Patient paid	$89 ($528)	$250 ($3590)	$98 ($71–$129)
Inpatient hospitalization	$5667 ($26,970)	$18,462 ($165,334)	$17,671 ($13,066–$23,292)
Plan paid	$5613 ($26,859)	$18,262 ($162,695)	$20,605 ($15,511–$26,816)
Patient paid	$55 ($461)	$201 ($3549)	$104 ($81–$131)
ED visit	$111 ($749)	$204 ($910)	$131 ($90–$180)
Plan paid	$107 ($738)	$200 ($906)	$138 ($99–186)
Patient paid	$4 ($76)	$4 ($25)	$2 ($2–$3)
Outpatient visit	$2058 ($14,467)	$3960 ($11,355)	$1365 ($721–$2123)
Plan paid	$2028 ($14,449)	$3914 ($11,255)	$1592 ($950–$2347)
Patient paid	$30 ($182)	$45 ($456)	$10 ($4–$16)

*ED*, emergency department; *irAE*, immune-related adverse event; *CTLA-4*, cytotoxic T-lymphocyte-associated protein 4; *PD-1*, programmed death-1; *PD-L1*, programmed death ligand-1.

^a^Regression model unable to converge.

**Table 4:** Unadjusted and adjusted means for all total medical costs

	Unadjusted		Diff	Adjusted		Diff
	Monotherapy	Combination therapy		Monotherapy	Combination therapy	
Total	$26,741	$67,877	$41,136	$36,976	$80,723	$43,747
Health plan paid	$26,354	$67,309	$40,955	$35,130	$83,573	$48,443
Patient paid	$387	$569	$182	$491	$643	$151

**Fig. 1:**
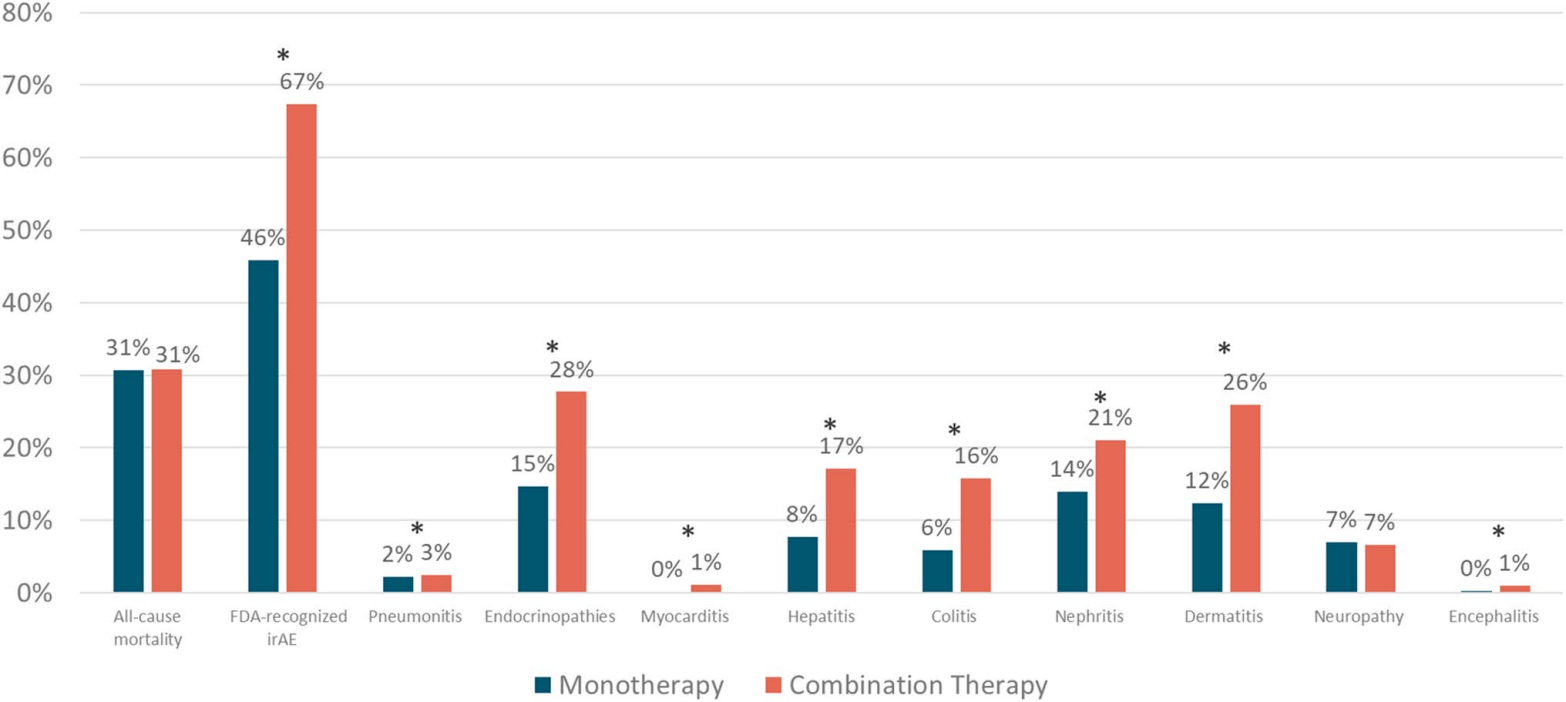
Unadjusted mortality and FDA immune-related adverse events. *p < 0.001

**Fig. 2:**
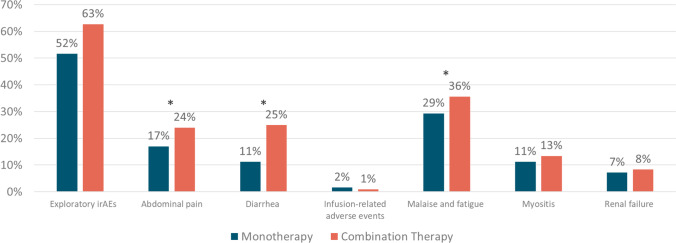
Unadjusted exploratory immune-related adverse events. *p < 0.001

The unadjusted FDA and exploratory irAEs are shown in Figs. 1 and 2, and the results of the adjusted Cox regression for incident irAE outcomes are presented in Table 2. The overall utilization and cost outcomes are presented in Tables 3 and 4.

## Discussion

To our knowledge, this is the first large retrospective study examining utilization and costs of ICI in the standard clinical practice setting. This study’s primary objective was to evaluate healthcare utilization and cost of mono and combination ICI therapy among the commercial and Medicare Advantage population up to 1-year post-index. We also compared rates of all-cause mortality and irAEs in this population.

A key element of this study was enumerating the incidence of irAEs in patients receiving both mono and combo therapy and assessing the differences. From other studies, such as published by Brown et al., showed that 61.8% experienced irAEs with ICI [6] and fatal irAEs occurred at 0.3–1.3% and often resulted in rapid clinical deterioration [7]. In our study, we observed these irAEs were a leading cause of toxicity in monotherapy: endocrinopathies, nephritis, dermatitis, hepatitis, and neuropathy and in combination therapy: endocrinopathies, dermatitis, nephritis, hepatitis, colitis, and neuropathy. As expected, it was observed that the combo arm had a greater rate of irAES. This was associated with an increase in all-cause hospitalizations, all-cause ED visits, and irAE-associated hospitalizations and ED visits. Our objective of the study was to capture important symptoms that patients experienced as well as more defined clinical diagnoses reflected in the claims. It is important to note that we used a broad definition of irAEs for the purposes of our study. This led to the identification of more irAEs than were reported in other single institution studies looking at chart-reviewed ED visits for patients undergoing ICI therapy [8, 9]. It is worth noting that the safety data concerning ICI therapy is limited to outpatients with good performance status. All-cause mortality was similar in both groups, which could be related to clinicians developing skills in identifying and treating toxicities earlier.

There is still much to understand about irAEs and multiple societal guidelines for irAE and ICI usage such as ASCO, ESMO, and MASCC are available to guide clinicians [10–12]. For example, it is not clear who is at highest risk for these toxicities, or why severe toxicities happen in certain populations. To develop and validate a prediction tool for irAEs, the SouthWest Oncology Cancer Research Network (SWOG) is currently enrolling solid tumor patients who are receiving ICI as mono or in combination with other ICIs (Immune Checkpoint Inhibitor Toxicity (I-CHECKIT).

This study is the first study reporting the healthcare utilization and cost in commercial and Medicare Advantage insured patients in the USA, which reflects the majority of clinical practice. Data from this study can support proper use of ICI in clinical practice and educate clinicians about the toxicity, care utilization, and cost.

Several limitations must be noted due to the retrospective and claims-based nature of our data. First, there were some missing data. For example, not all patients had performance status scores reported for their respective cancers. To address this, we used a claims-based algorithm as a proxy for ECOG status, which mapped well to patients who did have existing ECOG scores. We did not know the grade of tumor or stage of cancer, which may have affected selection of treatment and complicates the interpretation of all-cause mortality. ICD codes were used to identify cancers, irAEs, and comorbid conditions. There may have been errors or inaccuracies in coding. Moreover, we could not determine causal relationships between irAEs and use of ICIs. It is recognized that there are differences in reporting of various irAEs in our dataset compared to other publications. Our data reflects a large patient population across all types of practice settings, and diagnoses are based on coding of claims data. These methods reflect real-world practice. Some irAEs such as nephritis appear high and may also reflect an under appreciation of this potential toxicity in clinical practice. However, we tried to mitigate this by using incident, rather than prevalent irAEs. Finally, the study population consisted of patients with commercial or Medicare Advantage insurance in the USA and these data may not be generalizable to other populations such as the Medicaid population and outside of the USA.

## Conclusions

This large retrospective analysis of healthcare utilization and cost in commercial and Medicare Advantage patients receiving ICI in 1-year post-index period showed higher rates of irAEs, all-cause hospitalization and ED visits, irAE-related hospitalizations, and ED visits with combination ICI compared to monotherapy. These data provide useful estimates to help inform clinicians on the effects of combination therapy and for guideline development and application of relative clinical value frameworks in oncology. There are an increasing number of indications with combination ICI across multiple tumor types, and this study brings attention to the realities related to toxicity, utilization, and costs for clinicians who engage in shared decision-making with cancer patients. Based on published clinical trial data alone, it is far too easy to overlook the scope and importance of toxicities and costs for exciting new realms of cancer therapy.

## Supplementary Information

Below is the link to the electronic supplementary material.

Supplementary file1 (PDF 115 KB)

## Data Availability

We do not have full control of the primary data and thus, data are not available for sharing.

## Code availability

Code is not available for sharing due to use of proprietary software for data acquisition.
