# Supplementary material for: Immune checkpoint inhibitors: immune-related adverse events, healthcare utilization, and costs among commercial and Medicare Advantage patients
Source: Support Care Cancer. 2022 Jan 21;30(5):4019–26. doi: 10.1007/s00520-022-06826-9 (PMC8942885; doi:10.1007/s00520-022-06826-9)

Title: Immune checkpoint inhibitors: immune-related adverse events, healthcare utilization and costs among commercial and Medicare Advantage patients

Journal: Supportive Care in Cancer

Authors: Krishna S. Gunturu MD[1], Timothy T. Pham, PhD[2], Sonali Shambhu, MPH[2], Michael J. Fisch, MD[3], John J. Barron, PharmD[2], David Debono, MD[4]

1 Lahey Hospital and Medical Center, Burlington, MA 01805; 2 HealthCore, 123 Justison St, Suite 200, Wilmington, DE 19801 USA:3 AIM Specialty Health, Chicago, IL ; 4 Anthem Inc, 220 Virginia Ave, Indianapolis IN 46204.

Corresponding Author:

Lahey Hospital and Medical Center and
Lahey Health Cancer Institute,
Beth Israel Lahey Health
41 Mall Road, Burlington, MA 01805
781-744-8400
Krishna.S.Gunturu@lahey.org

Supplementary Online Content

eTable 1: FDA-recognized immune-related adverse events

irAE type	ICD-10 code
Pneumonitis	J70.2, J70.3, J70.4, J67.7, J84.09, J84.114, J84.115, J84.17, J84.89, J84.9
Endocrinopathies	E27.49, E27.40, E27.3, E06.4, E06.9, E06.1, E06.0, E09.xx, E23.1, E03.4, E03.2, E03.5, E03.8, E03.9, E05.01, E05.20, E05.81, E05.00, E05.31, E05.11, E05.21, E05.80, E05.90, E05.10, E05.30, E05.40, E05.41, E05.91
Myocarditis	I40.9, I41, I40.8, I40.1, I51.4
Hepatitis	K71.xx, K72.xx, K72.9x, K75.4x, K75.9x, R16.0x, R16.2x, R74.0x
Colitis	K51.40, K51.411, K51.412, K51.413, K51.414, K51.418, K51.419, K51.50, K51.511, K51.512, K51.513, K51.514, K51.518, K51.519, K51.80, K51.812, K51.813, K51.818, K51.914, K52.1, K52.3, K52.838, K52.839, K52.89, K52.9
Nephritis	N00.xx, N01.xx, N04.xx, N05.xx, N12.xx, N14.1x, N14.2x, N14.4x, N15.9x, N16.xx, N17.xx
Dermatitis	L29.xx, L49.xx, L50.xx, L51.xx, L52.xx, L53.8x, L53.9x, L95.xx, R21.xx
Neuropathy	G59, G63, G99.0, G90.9, G56.00, G56.01, G56.02, G56.03, G56.10, G56.11, G56.12, G56.13, G56.20, G56.21, G56.22, G56.23, G56.30, G56.31, G56.32, G56.33, G56.40, G56.41, G56.42, G56.43, G56.80, G56.81, G56.82, G56.83, G56.90, G56.91, G56.92, G56.93, G57.00, G57.01, G57.02, G57.03, G57.10, G57.11, G57.12, G57.13, G57.20, G57.21, G57.22, G57.23, G57.30, G57.31, G57.32, G57.33, G57.40, G57.41, G57.42, G57.43, G57.50, G57.51, G57.52, G57.53, G57.60, G57.61, G57.62, G57.63, G57.70, G57.71, G57.72, G57.73, G57.80, G57.81, G57.82, G57.83, G57.90, G57.91, G57.92, G57.93, G58.0, G58.7, G58.8, G58.9, G59, G61.82, G61.89, G61.9, G62.0, G62.2, G62.89, G62.9, G63
Encephalitis	G04.00, G04.30, G05.4, G04.39, G04.89, G05.3, G04.81, G04.91, G04.90

eTable 2: Exploratory immune-related adverse events

**irAE type**	**ICD-10 code**
Abdominal pain	R10.0, R10.1x, R10.3x, R10.8x, R10.9
Diarrhea	R19.7, K59.1
Infusion-related adverse events	T78.2x, T88.6x, T80.0x, T80.1x, T80.2x, T80.8x, T80.90x
Malaise and fatigue	R53.81, R53.83, R53.1
Myositis	G72.0, G72.9, M62.81, M62.82, M60.10, M60.111, M60.112, M60.119, M60.121, M60.122, M60.129, M60.131, M60.132, M60.139, M60.141, M60.149, M60.151, M60.152, M60.161, M60.162, M60.169, M60.171, M60.172, M60.18, M60.19, M60.80, M60.811, M60.812, M60.819, M60.821, M60.822, M60.829, M60.831, M60.832, M60.839, M60.841, M60.842, M60.849, M60.851, M60.852, M60.859, M60.861, M60.862, M60.869, M60.871, M60.872, M60.879, M60.88, M60.89, M60.9
Renal failure	I12.0, I13.10, I13.11, N18.1, N18.2, N18.3, N18.4, N18.5, N18.6, N18.9, N19, N25.0, Z49.01, Z49.02, Z94.0, Z99.2

eFigure 1. Flow chart of patient inclusion/exclusion

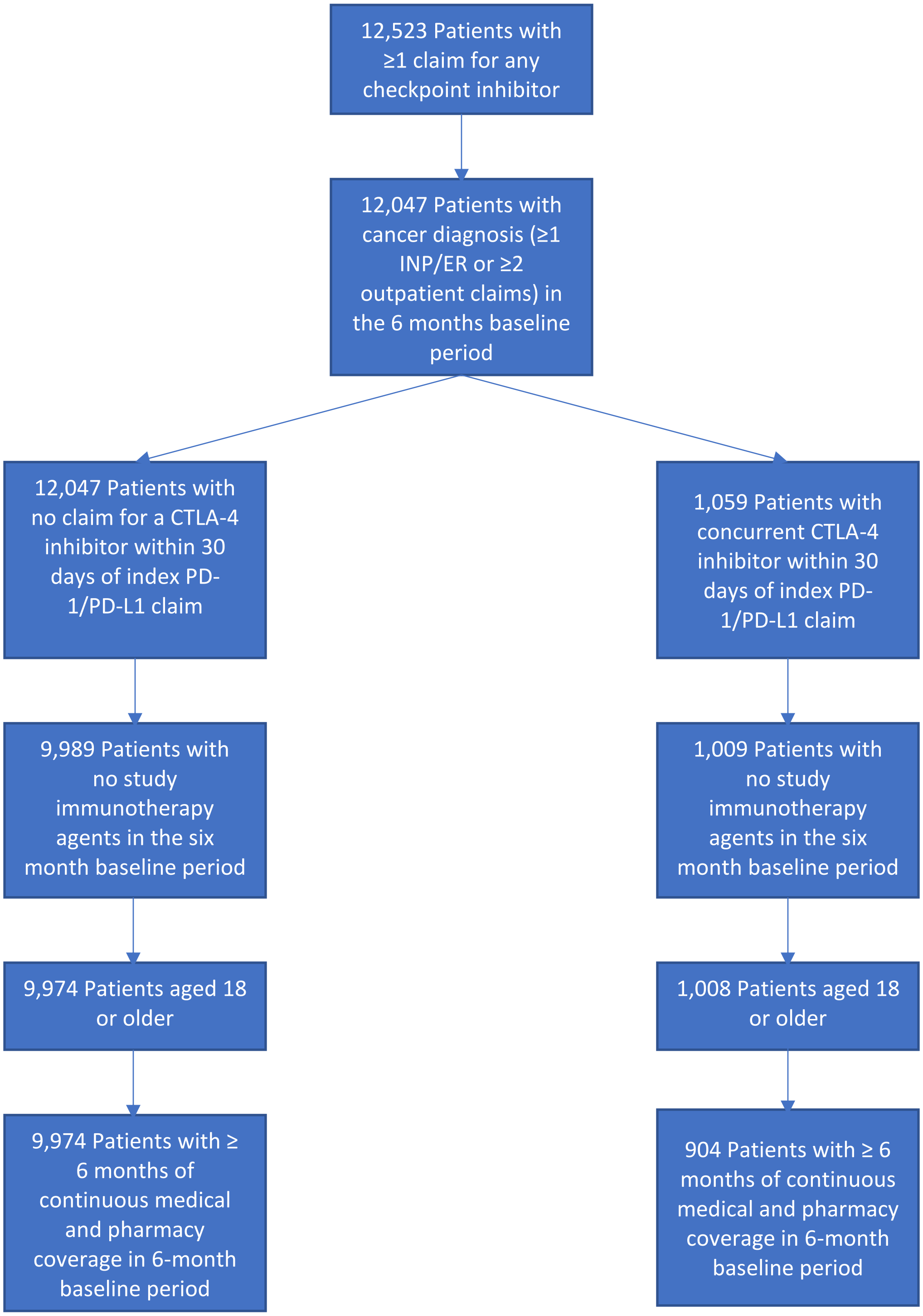

Supplement: Supplementary file 1 — Supplementary file1 (PDF 115 KB) [file 520_2022_6826_MOESM1_ESM.pdf]
